# C-C Motif Chemokine Ligand 2 (CCL2) Mediates Acute Lung Injury Induced by Lethal Influenza H7N9 Virus

**DOI:** 10.3389/fmicb.2017.00587

**Published:** 2017-04-04

**Authors:** Chengcai Lai, Keyu Wang, Zhongpeng Zhao, Liangyan Zhang, Hongjing Gu, Penghui Yang, Xiliang Wang

**Affiliations:** ^1^State Key Laboratory of Pathogens and Biosecurity, Beijing Institute of Microbiology and EpidemiologyBeijing, China; ^2^Beijing 302 HospitalBeijing, China

**Keywords:** lethal influenza H7N9 virus, acute lung injury (ALI), CCL2, experimental mouse model, influenza H7N9 Hb01 virus

## Abstract

An avian-origin influenza A (H7N9) virus was a cause for concern in China in the spring of 2013. Most H7N9 infections resulted in acute respiratory distress syndrome (ARDS), which is a severe form of acute lung injury (ALI) that contributes to morbidity and mortality. In this study, we induced viral ALI by infecting wild-type and CCL2-deficient mice with influenza H7N9 virus. The results suggested a close association between C-C motif chemokine ligand 2 (CCL2) expressions and ALI induced by a lethal H7N9 virus strain (A/Hebei/01/2013). Elevated CCL2 levels were also detected in confirmed human cases of H7N9 and the bronchoalveolar lavage fluid (BALF) of H7N9-infected mice. Moreover, CCL2 was overexpressed in the lung tissue of infected mice. More importantly, CCL2 deficiency ameliorated H7N9-induced ALI in mice as determined by weight loss, survival rate, the wet:dry ratio of the lung, and pathology. Taken together, our findings demonstrate that CCL2 is essential for H7N9 virus infection and thus that it is a potential therapeutic target for influenza.

## Introduction

The influenza pandemic caused by avian influenza A (H7N9) was a cause for concern in eastern China beginning in March 2013(Watanabe et al., 2013; Zhu et al., 2013). H7N9 infection resulted in fever and cough, acute lung injury (ALI), and acute respiratory distress syndrome (ARDS), which frequently required admission to an intensive care unit (ICU); moreover, it was associated with considerable morbidity and mortality (Chen et al., 2013). As of July 19, 2016, a total of 793 cases had been reported, including 319 deaths (Xiang et al., 2017).

Most infected patients have exhibited excessive cytokine and chemokine production, which could result in hypercytokinemia and is associated with disease severity (Chen et al., 2013; Chi et al., 2013; Huang et al., 2014; Guo et al., 2015). Hypercytokinemia, also known as a “cytokine storm,” is an early indicator of H7N9-induced ALI. However, the relationship between cytokine production and the pathogenicity of H7N9 is unclear. C-C motif chemokine ligand 2 (CCL2), also known as monocyte chemoattractant protein (MCP)-1, binds to the chemokine receptors CCR2 and CCR5, and is an important regulator of monocyte/macrophage trafficking during infection or in the presence of inflammation (Takahashi et al., 2003; Herold et al., 2006; Yadav et al., 2010). Previous studies have shown that CCL2 plays a crucial role in influenza virus H1N1 infection (Maelfait et al., 2016). Although increased expression of CCL2 during influenza infection has been well-documented (Zhou et al., 2013; Guo et al., 2015), the effects of the CCL2/CCR2 pathway on the pathogenesis of infection are controversial (Dawson et al., 2000; Narasaraju et al., 2010; Maelfait et al., 2016). Thus, we determined the effects of CCL2 on the severity of H7N9-induced ALI in an experimental mouse model. We hypothesized that CCL2 participates in the pathogenesis of the fulminant phase of H7N9-induced ALI and may be a useful therapeutic target.

## Materials and methods

### Animals and virus

Specific-pathogen-free (SPF), 4-week-old, wild-type C57BL/6 (B6) mice were obtained from the Experimental Animal Center of the Academy of Military Medical Sciences (AMMS), Beijing, China. SPF, 4-week-old, CCL2-deficient (CCL2^−/−^) mice (Jackson Laboratories) were housed at the animal facility of the Beijing Institute of Microbiology and Epidemiology in accordance with institutional guidelines. The influenza A H7N9 virus strain A/Hebei/01/2013 (Hb01) was initially isolated from a throat-swab specimen of a confirmed H7N9-infected patient in Beijing and propagated by inoculation into SPF 9- to 11-day-old embryonated eggs by the allantoic route. The accession numbers of Hb01 in the GISAID database are EPI509120–EPI509127.

All experimental protocols involving animals were approved by the Institutional Animal Care and Use Committee of the AMMS (ID: SYXK2012-05), and all procedures conformed to the relevant regulatory standards. All experiments involving live virus were performed in an approved biosafety level 3 facility.

### Experimental mouse models of lethal H7N9-induced acute lung injury

SPF, 4-week-old B6 mice were anesthetized with 40 mg/kg 1% (w/v) pentobarbital sodium and received 10^3^ of the 50% tissue culture infectious dose (TCID_50_) of Hb01 virus in 20 μL allantoic fluid (AF) or an identical volume of control AF intranasally. The survival rate, changes in body weight, histological parameters, and acute lung edema (wet:dry ratio) were evaluated as described previously (Li et al., 2012). Lung tissues were collected in 10% neutral-buffered formalin and stained with hematoxylin and eosin (H&E). The number of infiltrating inflammatory cells was counted and presented as the number of cells per 200× field. The analyses were performed in a blinded fashion.

### Cytokine and chemokine measurement

Serum and bronchoalveolar lavage fluid (BALF) samples were collected from the infected mice at indicated time points. For cytokine and chemokine measurements, serum and BALF samples were processed using the Bio-Plex Mouse Cytokine 23-Plex instrument (Bio-Rad Laboratories) following the manufacturer's instructions, and array analyses were performed using a Bio-Plex Protein Array system (Bio-Rad Laboratories).

### Measurement of patient serum levels of CCL2

The CCL2 levels in serum samples from 29 human patients infected with H7N9 were measured using a Bio-Plex Human Cytokine 25-Plex array (Bio-Rad Laboratories). Subjects provided written informed consent for participation in this study.

### Confocal fluorescence microscopy

The lung tissues of mice were harvested, fixed in 10% formalin, and processed for immunohistochemistry. A rabbit anti-mouse CCL2 polyclonal antibody (Cell Signaling Technology, USA) and goat anti-rabbit IgG-Alexa Fluor 488 (Bioss, Beijing) antibody were used for immunohistochemistry. Nuclei were stained with DAPI (Vector Labs), and visualized using a confocal laser scanning microscope (FV1000, Olympus).

### Statistical analyses

All data are presented as means ± SEM. Statistical analyses were performed using Student's *t*-tests. Survival data were analyzed using the Kaplan-Meier method, and single time points were subjected to analysis of variance (ANOVA). All analyses were performed using Instat software (version 5.0, GraphPad). A value of *p* < 0.05 was taken to indicate statistical significance.

## Results

### Mice infected with influenza H7N9 virus Hb01 develop severe ALI

**Figure 1:**
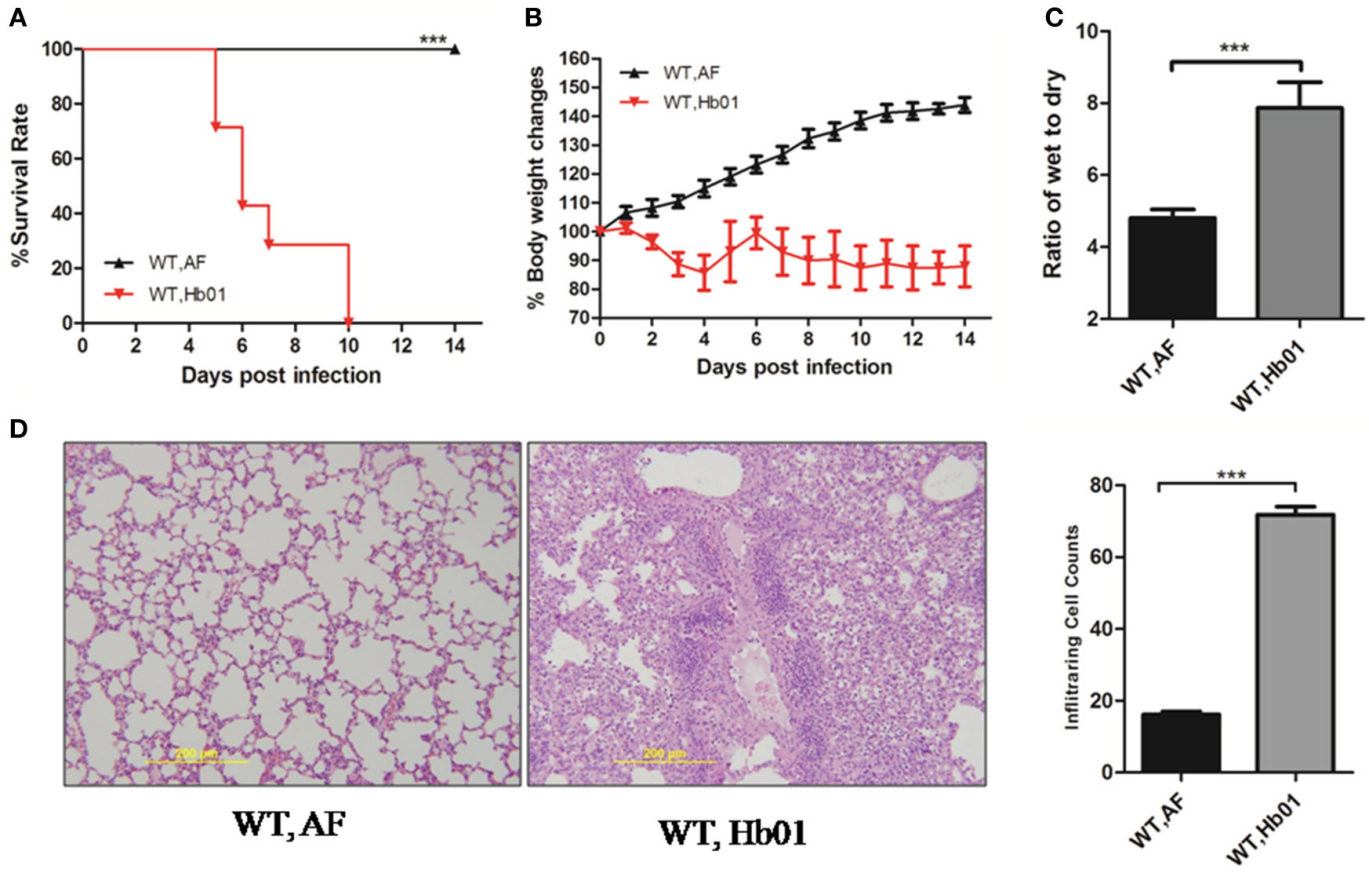
**Influenza H7N9 Hb01-induced ALI in mice**. Four-week-old B6 mice were anesthetized and inoculated with 10^3^ TCID_50_ Hb01 virus in 20 μL allantoic fluid (AF) or an identical volume of control AF. Survival rates **(A)** and body weight changes **(B)** of B6 mice (*n* = 10) inoculated with AF and Hb01. Wet:dry ratios **(C)** of mouse lung tissues (*n* = 6) at 5 DPI. H&E-stained images **(D)** and infiltrating cell counts (*n* = 100 fields) of the lung tissues of infected mice at 5 DPI (magnification = 200×). ^***^p < 0.001.

To evaluate the pathogenicity of influenza H7N9 virus Hb01, 4-week-old C57BL/6 mice were inoculated intranasally (i.n.) with 10^3^ TCID_50_ Hb01. The mice began to die at 5 days post-infection (DPI), and almost all had died by 10 DPI (Figure 1A). All of the infected mice showed significant weight loss of up to 30%, particularly at 9–11 DPI (Figure 1B). Moreover, lung edema, defined as the wet:dry ratio of lung tissue, was observed following Hb01 infection (Figure 1C). In addition, we evaluated histological changes in lung tissues and found that histopathology worsened significantly at 5 DPI (Figure 1D). Multiple leukocyte infiltrations were detected in lung tissue, but there were no significant histological changes in the intestine, brain, or kidney tissues (Figure S1). By contrast, there were less numbers of leukocytes in the BALF of CCL2-deficient mice (data not shown). Taken together, these data indicate that 4-week-old C57 mice infected with Hb01 have potential as an animal model of severe 2013 influenza H7N9 infection.

### Cytokine responses in mice infected with influenza H7N9 Hb01 virus

**Figure 2:**
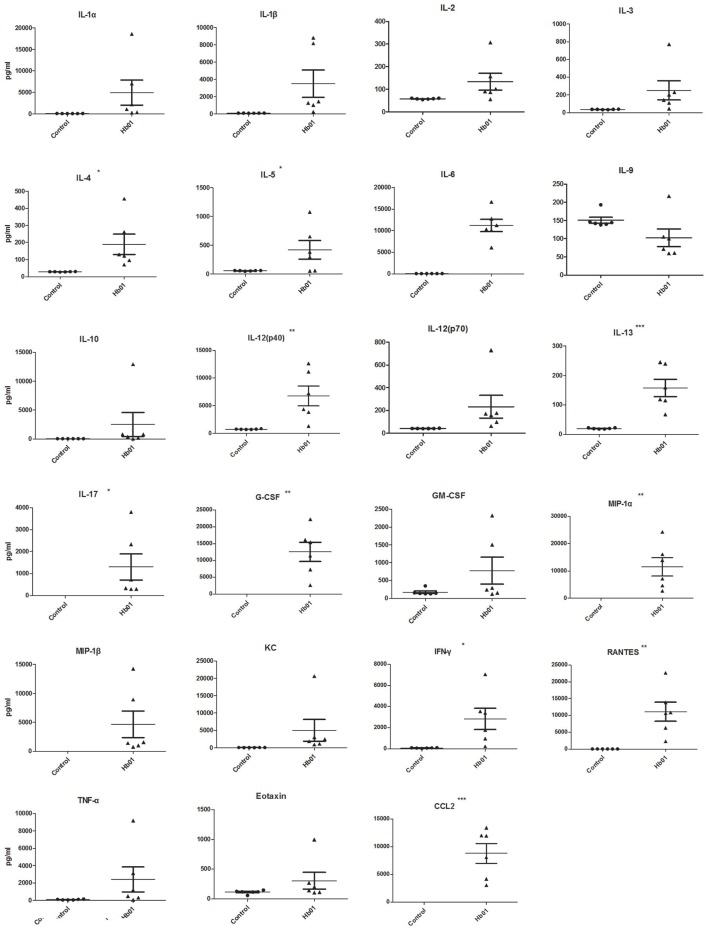
**Cytokine and chemokine levels in BALF of Hb01-infected mice**. Cytokine and chemokine concentrations in mouse BALF (*n* = 6) at 5 days after inoculation with 10^3^ TCID_50_ Hb01 or an identical volume of allantoic fluid were determined using a Mouse Cytokine 23-Plex Array (Bio-Rad Laboratories). ^*^p < 0.05, ^**^p < 0.01, and ^***^p < 0.001.

Most patients infected with influenza H7N9 virus exhibit hypercytokinemia, which leads to influenza virus-induced ALI (Zhou et al., 2013). The levels of 23 cytokines and chemokines in mouse BALF and serum at 5 DPI were investigated; IL-1α, IL-2, MIP-1β, and GM-CSF levels were significantly upregulated in serum but were unchanged in BALF. In addition, IL-4, IL-5, and IL-13 levels were upregulated in BALF but were unchanged in serum. IL-12(p40), IL-17, G-CSF, MIP-1α, IFN-γ, RANTES, and CCL2 levels were significantly upregulated in serum and BALF upon H7N9 challenge (Figure 2, Figure S2). Strikingly, CCL2 levels in both BALF and serum remained high following H7N9 infection.

### CCL2 is significantly upregulated following influenza H7N9 virus infection

**Figure 3:**
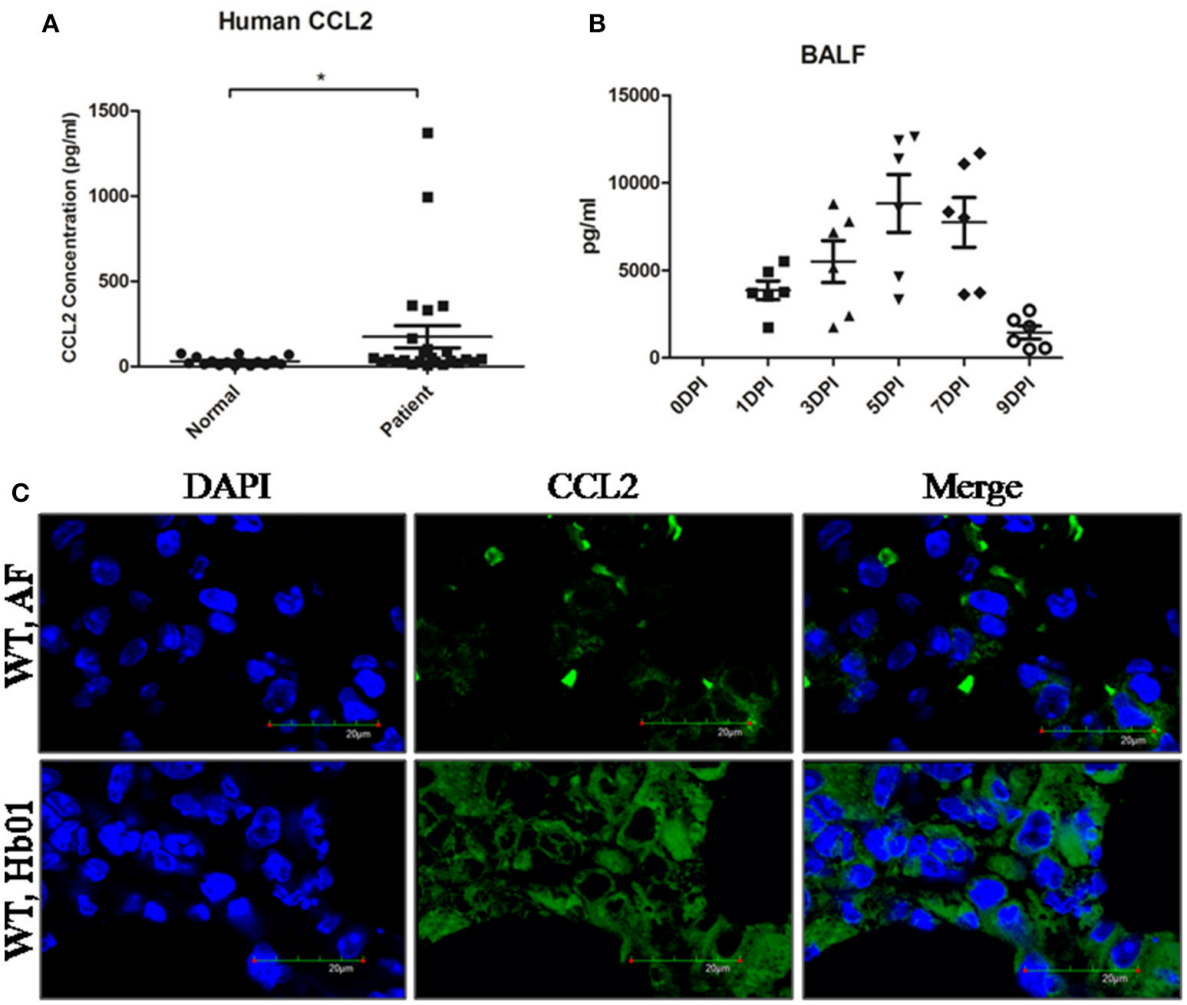
**CCL2 levels were significantly increased in the serum of H7N9-infected patients and the BALF and lung tissues of Hb01-infected mice**. The serum concentration of CCL2 of H7N9-infected patients (*n* = 29) was determined using a Bio-Plex Human Cytokine 25-Plex array (Bio-Rad Laboratories) **(A)**. Four-week-old B6 mice were anesthetized and inoculated with 10^3^ TCID_50_ Hb01 virus. The level of CCL2 in BALF **(B)** was assayed using a Mouse Cytokine 23-Plex Array (Bio-Rad Laboratories) at the indicated time points. **(C)** The CCL2 level in mouse lung tissue at 5 days after Hb01 infection was evaluated by fluorescence microscopy. CCL2 was stained with Alexa Flour 488 (green) and nuclei were strained with DAPI (blue). ^*^p < 0.05.

During the 2013 H7N9 influenza pandemic, we recruited 29 patients (Table S1) who were PCR-positive for H7N9, together with 17 healthy individuals, from Quanzhou Hospital, Beijing 302 Hospital, and Beijing Chaoyang Hospital. The plasma concentrations of CCL2 of the confirmed H7N9-infected patients were significantly elevated compared to the control group (Figure 3A), confirming previous reports (Zhou et al., 2013; Guo et al., 2015). Similarly, CCL2 was significantly upregulated beginning at 1 DPI in mouse BALF (Figure 3B) and serum (Figure S3), and remained high in BALF up to 9 DPI (Figure 3B); indeed, it remained high in mouse serum following Hb01 infection (Figure S3). Furthermore, the lung tissues of mice exhibited a high level of CCL2 at 5 DPI (Figure 3C). Thus, we hypothesized that CCL2 might play a critical role in the host response to lethal influenza H7N9 virus infection.

### CCL2-deficient mice exhibit reduced severity of H7N9 virus-induced ALI

**Figure 4:**
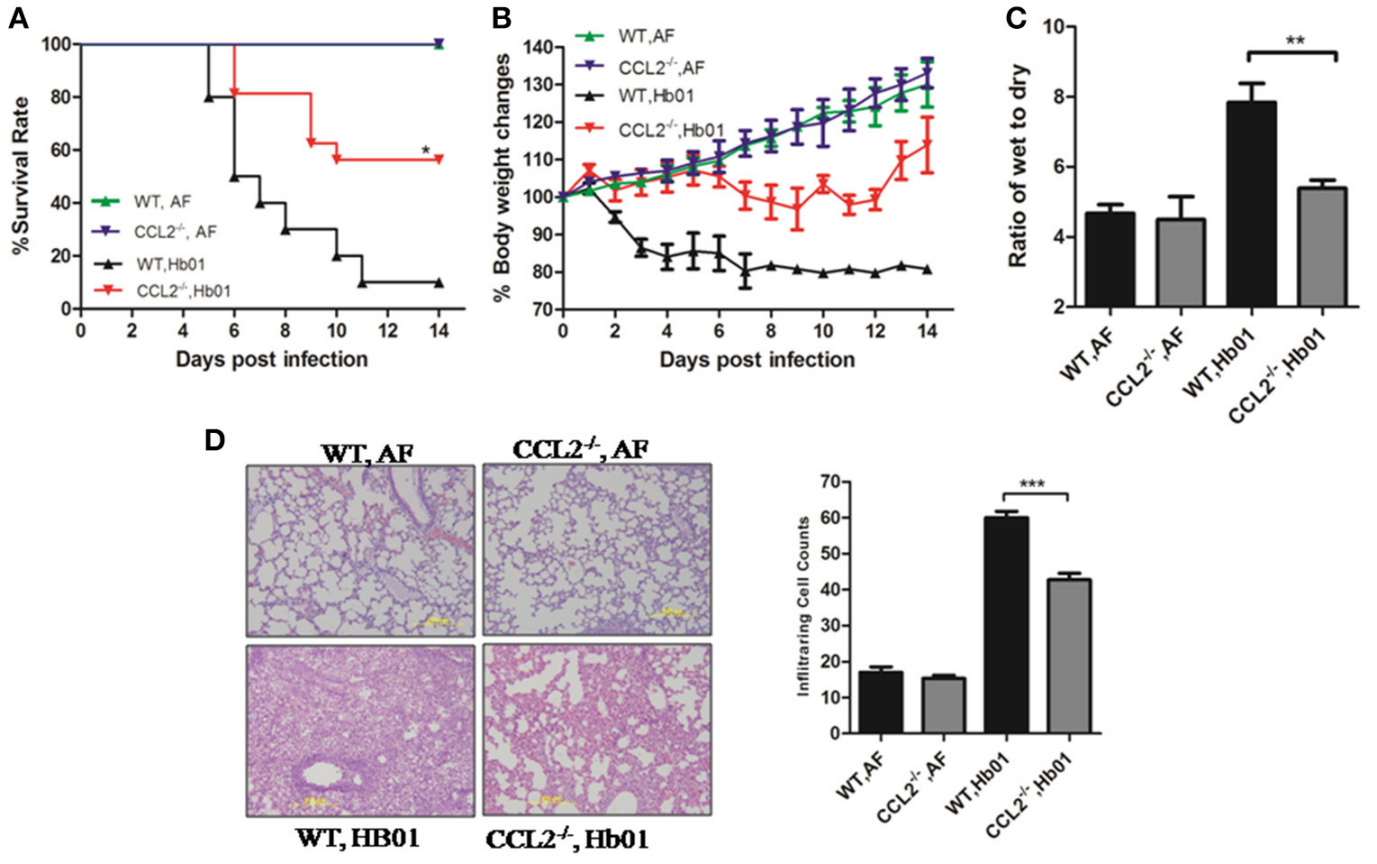
**CCL2^**−/−**^ mice exhibited reduced severity of lung injury**. Four-week-old CCL2^−/−^ and wild-type B6 mice were inoculated with 10^3^ TCID_50_ Hb01 virus. Survival rates **(A)** and body weight changes **(B)** of wild-type and CCL2^−/−^ mice (*n* = 10) were monitored for 2 weeks after Hb01 challenge. Wet:dry ratios **(C)** of lung tissues (*n* = 6) at 5 DPI. HE-stained images **(D)** and infiltrating cell counts (*n* = 100 fields) of lung tissues at 5 DPI (magnification = 200×). ^*^p < 0.05, ^**^p < 0.01, and ^***^p < 0.001.

To elucidate the functional role of CCL2 in ALI, CCL2-deficient mice were infected with lethal influenza H7N9 virus Hb01; survival was significantly improved compared to infected wild-type mice (Figure 4A). Rapid and irreversible weight loss (>20%) occurred in infected wild-type control mice but not in CCL2^−/−^ mice following infection (Figure 4B). In addition, lung edema was significantly ameliorated in infected CCL2^−/−^ mice (Figure 4C). Moreover, lung histopathology was significantly improved and leukocyte infiltration of lung tissue was significantly ameliorated in CCL2^−/−^ mice (Figure 4D). No pathologic changes were evident in the kidney, intestine, or brain tissues of Hb01-infected mice (Figure S1). These results confirm the important role of CCL2 in H7N9-induced ALI, and suggest that this factor is a potential therapeutic target.

## Discussion

H7N9-infected patients tend to have longer hospital stays and a higher mortality rate than H1N1-infected patients (Chen et al., 2013). Moreover, influenza H7N9 virus is more pathogenic to mice and ferrets than is seasonal influenza virus (Belser et al., 2013; Watanabe et al., 2013; Yum et al., 2015). For example, A/Anhui/01/H7N9/2013 (H7N9) induces severe respiratory disease in mice but is not lethal (Zhao et al., 2014), suggesting that H7N9 virus strains exhibit various levels of virulence in animal models. The role of CCL2 in influenza infection has been investigated, but the findings are controversial (Dawson et al., 2000; Narasaraju et al., 2010; Maelfait et al., 2016). Elevated CCL2 levels in patients infected with H7N9 virus have also been documented (Zhou et al., 2013; Guo et al., 2015). To the best of our knowledge, this is the first report of the impact of CCL2 deficiency in H7N9-infected mice.

Influenza H7N9 patients die not due to the infection itself but from ARDS caused by a cytokine storm, which is defined as a release of excessive levels of more than 150 inflammatory mediators (cytokines, oxygen free radicals, and coagulation factors). Our data indicate that the levels of vital proinflammatory cytokines and chemokines (including IL-12(p40), IL-17, G-CSF, MIP-1α, IFN-γ, and RANTES) were significantly increased in the BALF and serum of Hb01-virus infected mice. More importantly, the levels of cytokines and chemokines in BALF were higher than those in serum, likely because the lung is the target organ of influenza virus infection.

CCL2 is significantly up-regulated during H1N1, H5N1, and H7N9 infections (Li et al., 2012; Zhou et al., 2013; Lai et al., 2014). In addition, CCL2 plasma levels could reflect the severity of disease and predict fatal outcomes in patients infected with H7N9 (Diao et al., 2014; Qian et al., 2014; Guo et al., 2015). In this study, CCL2 levels were elevated in mouse BALF, serum, and lung tissues following H7N9 infection, in agreement with the increased levels in serum samples of confirmed H7N9-infected patients. In addition, Dawson et al. and other groups have reported that amelioration of CCL2 or CCR2 deficiency could protect mice from H1N1 infection (Dawson et al., 2000; Kok et al., 2012; Maelfait et al., 2016). However, CCL2 deficiency or CCL2 antibody treatment enhances damage and impedes repair of the alveolar epithelium after infection with a sublethal dose of influenza A virus or mouse-adapted human influenza virus (Dessing et al., 2007; Narasaraju et al., 2010). Of note, we investigated the role of CCL2 in ALI induced by H7N9 virus; CCL2 deficiency protected mice from lethal influenza H7N9 virus infection. CCL2^−/−^ mice showed less weight loss, a lower mortality rate, improved lung edema, and ameliorated histopathological changes post-infection.

The innate immune response acts as the first line of defense against pathogenic agents. Previous studies have revealed that innate immunity is essential for H5N1-induced ALI (Sun et al., 2012). Innate immunity plays a key role in resistance to influenza infection, but an excessive response is harmful to the host. Infiltration of inflammatory cells, particularly monocytes/macrophages, in the lung leads to the generation of a cytokine storm. Similarly, in another experiment, we found that Rag 1-knockout mice function in the course of lethal H7N9 induced-ALI in mice, indicating that the innate immune response plays a crucial role during influenza H7N9 virus infection (data not shown here). In addition, the viral titer in CCL2-null mice, and the effects of anti-CCL2 therapy of wild-type mice subjected to H7N9 infection, warrant further investigation. Such studies will likely confirm that CCL2 is a crucial mediator of the inflammatory response during H7N9 infection or is it affecting in some way the viral burden. We believe that clinical intervention using an appropriate immune modulator combined with antiviral treatment would benefit H7N9 patients.

In conclusion, mice infected with influenza H7N9 Hb01 virus exhibited severe ALI and other symptoms typical of influenza. Our data demonstrate an association between CCL2 expression and a severe form of ALI induced by H7N9 infection in mice. Therefore, CCL2 is a pivotal mediator in H7N9 infection and should be considered a promising therapeutic target.

## Author contributions

Conceived and designed the experiments: XW, PY. Performed the experiments: CL, KW. Analyzed the data: CL, HG, ZZ, LZ. Contributed reagents/materials/analysis tools: CL. Wrote the paper: CL, PY, XW.

### Conflict of interest statement

The authors declare that the research was conducted in the absence of any commercial or financial relationships that could be construed as a potential conflict of interest.

## Supplementary material

Figure S1 **HE-stained images of the brain, kidney, and intestine of Hb01-infected mice**. Four-week-old wild-type B6 and CCL2^−/−^ mice were inoculated with control AF or 10^3^ TCID_50_ Hb01 virus. The brain, kidney, and intestine of mice were harvested at 5 DPI and subjected to histopathological analysis (magnification = 200×).

Figure S2 **Serum levels of cytokines and chemokines of Hb01-infected mice**. Mouse Serum levels of cytokines and chemokines in mice (*n* = 6) at 5 days after inoculation with 10^3^ TCID_50_ Hb01 or an identical volume of allantoic fluid were determined using a Mouse Cytokine 23-Plex Array (Bio-Rad Laboratories). ^*^*p* < 0.05, ^**^*p* < 0.01, and ^***^*p* < 0.001.

Figure S3 **Serum levels of CCL2 in Hb01-infected mice**. Four-week-old B6 mice were anesthetized and inoculated with 10^3^ TCID_50_ Hb01 virus. Serum levels of CCL2 (*n* = 6) were determined using a Mouse Cytokine 23-Plex Array (Bio-Rad Laboratories) at the indicated time points.

Table S1 **Basic characteristics of patient recruited**.
