# Supplementary figures and images for: C-C Motif Chemokine Ligand 2 (CCL2) Mediates Acute Lung Injury Induced by Lethal Influenza H7N9 Virus
Source: Front Microbiol. 2017 Apr 4;8:587. doi: 10.3389/fmicb.2017.00587 (PMC5379033; doi:10.3389/fmicb.2017.00587)

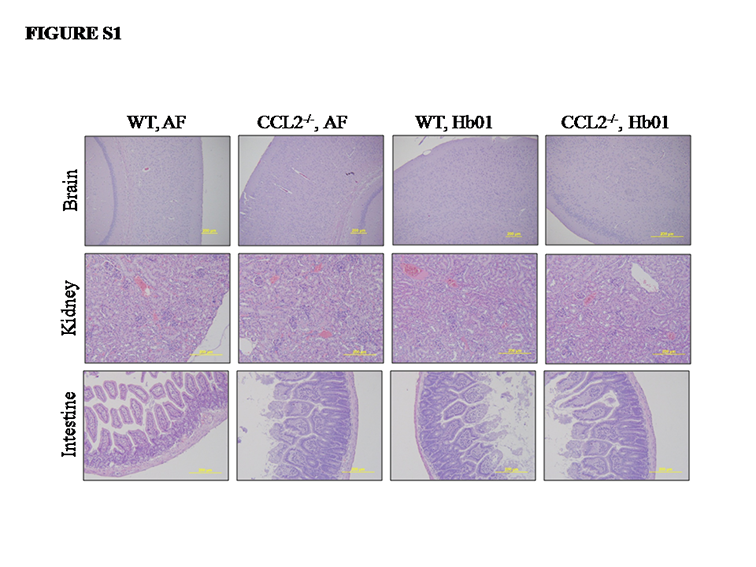

Supplement: Figure S1 — HE-stained images of the brain, kidney, and intestine of Hb01-infected mice. Four-week-old wild-type B6 and CCL2−/− mice were inoculated with control AF or 103 TCID50 Hb01 virus. The brain, kidney, and intestine of mice were harvested at 5 DPI and subjected to histopathological analysis (magnification = 200×). [file Image1.TIF]

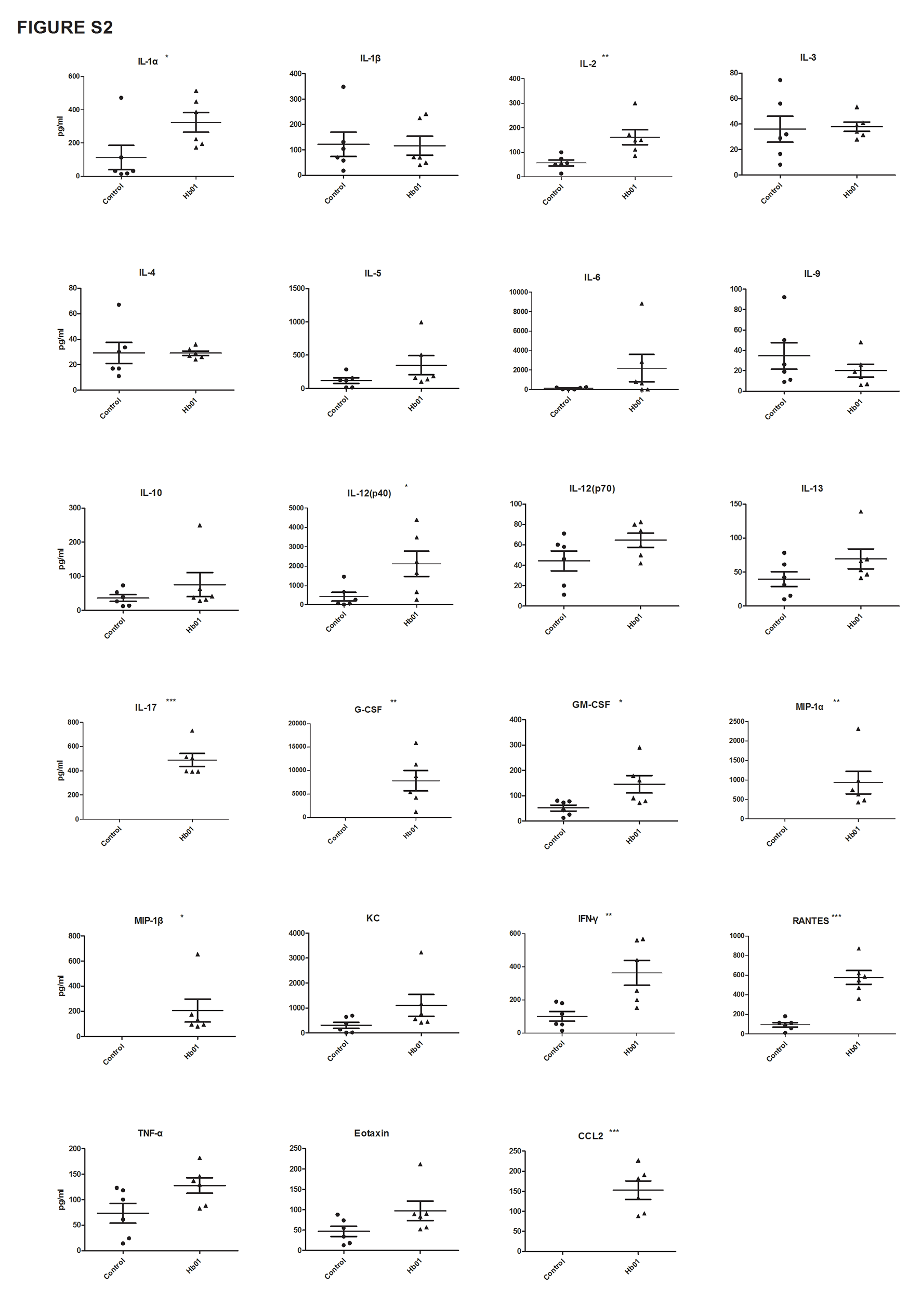

Supplement: Figure S2 — Serum levels of cytokines and chemokines of Hb01-infected mice. Mouse Serum levels of cytokines and chemokines in mice (n = 6) at 5 days after inoculation with 103 TCID50 Hb01 or an identical volume of allantoic fluid were determined using a Mouse Cytokine 23-Plex Array (Bio-Rad Laboratories). *p < 0.05, **p < 0.01, and ***p < 0.001. [file Image2.TIF]

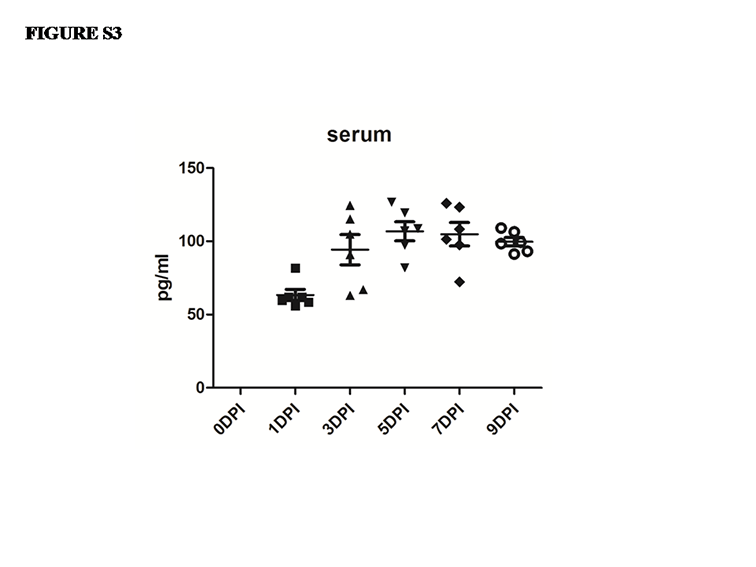

Supplement: Figure S3 — Serum levels of CCL2 in Hb01-infected mice. Four-week-old B6 mice were anesthetized and inoculated with 103 TCID50 Hb01 virus. Serum levels of CCL2 (n = 6) were determined using a Mouse Cytokine 23-Plex Array (Bio-Rad Laboratories) at the indicated time points. [file Image3.TIF]
